# Neurocognitive Outcomes in Pediatric Patients Following Brain Irradiation

**DOI:** 10.3390/cancers13143538

**Published:** 2021-07-15

**Authors:** Katharina Weusthof, Peggy Lüttich, Sebastian Regnery, Laila König, Denise Bernhardt, Olaf Witt, Klaus Herfarth, Andreas Unterberg, Christine Jungk, Benjamin Farnia, Stephanie E. Combs, Jürgen Debus, Stefan Rieken, Semi Harrabi, Sebastian Adeberg

**Affiliations:** 1 Department of Radiation Oncology, Heidelberg University Hospital, 69120 Heidelberg, Germany; katharina.weusthof@med.uni-heidelberg.de (K.W.); sebastian.regnery@med.uni-heidelberg.de (S.R.); laila.koenig@med.uni-heidelberg.de (L.K.); klaus.herfarth@med.uni-heidelberg.de (K.H.); juergen.debus@med.uni-heidelberg.de (J.D.); semi.harrabi@med.uni-heidelberg.de (S.H.); 2 Department of Radiation Oncology, Heidelberg Institute of Radiation Oncology (HIRO), 69120 Heidelberg, Germany; 3 Section of Pediatric Brain Tumors, Department of Pediatric Oncology, Hematology and Immunology, University Medical Center for Children and Adolescents, 69120 Heidelberg, Germany; peggy.luettich@med.uni-heidelberg.de (P.L.); olaf.witt@med.uni-heidelberg.de (O.W.); 4 National Center for Tumor Diseases (NCT), 69120 Heidelberg, Germany; 5 Department of Radiation Oncology, Heidelberg Ion-Beam Therapy Center (HIT), 69120 Heidelberg, Germany; 6 Department of Radiation Oncology, Technische Universität München, 81675 München, Germany; denise.bernhardt@tum.de (D.B.); stephanie.combs@tum.de (S.E.C.); 7 Department of Radiation Sciences (DRS), Institut für Innovative Radiotherapie (iRT), Helmholtz Zentrum München, 85764 Neuherberg, Germany; 8 Translational Program, Hopp Children’s Cancer Center at NCT Heidelberg (KiTZ), 69120 Heidelberg, Germany; 9 Clinical Cooperation Unit Radiation Oncology, German Cancer Research Center (DKFZ), 69120 Heidelberg, Germany; 10 German Cancer Consortium (DKTK), Partner Site Heidelberg, German Cancer Research Center (DKFZ), 69120 Heidelberg, Germany; 11 Department of Neurosurgery, Heidelberg University Hospital, 69120 Heidelberg, Germany; andreas.unterberg@med.uni-heidelberg.de (A.U.); christine.jungk@med.uni-heidelberg.de (C.J.); 12 Department of Radiation Oncology, Sylvester Comprehensive Cancer Center, University of Miami, Miami, FL 33136, USA; benjaminfarnia@msn.com; 13 Department of Radiation Oncology, University Hospital Goettingen, 37075 Goettingen, Germany; stefan.rieken@med.uni-goettingen.de

**Keywords:** pediatric brain tumor, proton irradiation, photon irradiation, treatment modality comparison, neuropsychological assessment, neurocognitive function

## Abstract

**Simple Summary:**

Since inception, radiation therapy in pediatric brain tumor patients has been associated with neurocognitive decline and persistent impairment. Recently published studies suggest improved cognitive outcomes with advanced radiation techniques due to increased conformality. Our study compares different treatment modalities through longitudinal assessment of seven neurocognitive domains as well as examining the overall effect on scholastic performance in long-term follow-up more than four years after treatment. Comprehensive data were available due to interdisciplinary cooperation of pediatric neuro-oncologists, radiation oncologists, neurosurgeons, and clinical psychologists. To our knowledge, only a few such detailed testing results have been published, allowing a more granular examination of neurocognitive outcomes rather than basic IQ testing. Our study was able to show no differences in performance after adding modern conformal proton or photon radiation therapy to surgery compared to surgery alone. We could rule out radiation therapy as severe confounding factor in neurocognitive decline after brain tumor treatment in our study.

**Abstract:**

Advanced radiation techniques can reduce the severity of neurocognitive sequelae in young brain tumor patients. In the present analysis, we sought to compare neurocognitive outcomes after proton irradiation with patients who underwent photon radiotherapy (RT) and surgery. Neurocognitive outcomes were evaluated in 103 pediatric brain tumor patients (proton RT *n* = 26, photon RT *n* = 30, surgery *n* = 47) before and after treatment. Comparison of neurocognitive outcomes following different treatment modalities were analyzed over four years after treatment completion. Longitudinal analyses included 42 months of follow-up after proton RT and 55 months after photon RT and surgery. Neurocognitive assessment included standardized tests examining seven domains. A comparison of neurocognitive outcomes after RT (proton and photon with >90% additional surgery) and surgery showed no significant differences in any neurocognitive domain. Neurocognitive functioning tests after proton RT failed to identify alterations compared to baseline testing. Long-term follow up over four years after photon RT showed a decrease in non-verbal intelligence (−9.6%; *p* = 0.01) and visuospatial construction (−14.9%; *p* = 0.02). After surgery, there was a decline in non-verbal intelligence (−10.7%; *p* = 0.01) and processing speed (14.9%; *p* = 0.002). Differences in neurocognitive outcomes between RT and surgical cohorts in direct intermodal comparison at long-term follow-up were not identified in our study, suggesting that modern radiation therapy does not affect cognition as much as in the past. There were no alterations in long-term neurocognitive abilities after proton RT, whereas decline of processing speed, non-verbal intelligence, and visuospatial abilities were observed after both photon RT and surgery. Domains dependent on intact white matter structures appear particularly vulnerable to brain tumor treatment irrespective of treatment approach.

## 1. Introduction

Tumors of the central nervous system are frequently encountered in pediatric oncology, accounting for approximately 20% of all childhood malignancies [1,2]. Thanks to improved multimodality therapy, survival rates have increased over the last several decades. As a result, preservation of quality of life and neurocognitive function plays an increasingly important role in the management of these malignancies. Trials utilizing less conformal radiation (RT) techniques [3,4,5,6,7,8] show a neurocognitive decline after treatment. Recent studies, however, suggest that increased RT conformality, achieved via IMRT (Intensity Modulated Radiation Therapy) or proton RT [9,10] lead to fewer long-term deficits in neurocognitive function [7,11,12,13,14]. The primary aim of the present study is to compare the neurocognitive outcome of pediatric brain tumor patients after multimodal brain tumor therapy, especially comparing the main risk factors for neurocognitive decline: proton RT, photon RT, or surgery alone. We investigated differences in the longitudinal development of numerous neurocognitive domains after treatment and sought to elucidate the vulnerability of specific domains by neuroanatomic deficits.

## 2. Materials and Methods

### 2.1. Patient and Treatment Characteristics

**Figure 1:**
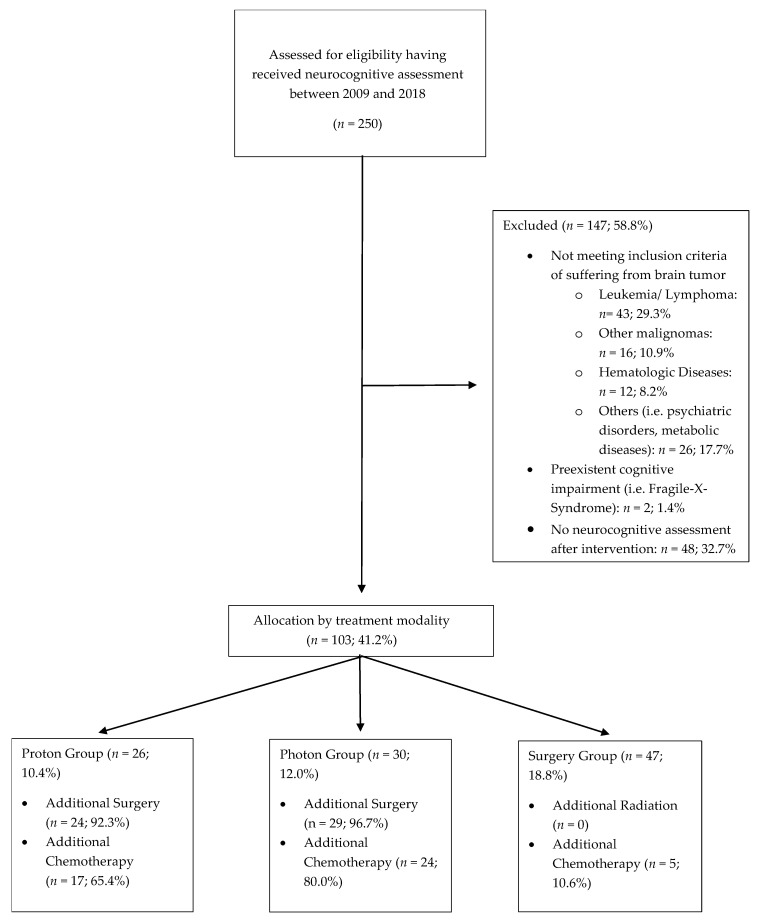
Consort Diagram: Neurocognitive Assessment of Pediatric Brain Tumor Patients.

Pediatric patients treated at our hospital from 2009 to 2018 who received at least one neurocognitive assessment were eligible for study inclusion, independent of treatment modality. Within this timeframe, 103 patients received brain tumor and neuropsychological treatment. Of this cohort, 26 (13.1%) underwent proton radiotherapy, 30 patients underwent photon irradiation, and 47 patients received surgical resection without further adjuvant treatment. Given that RT alone is currently not the standard of care, the majority of patients treated with proton or photon irradiation underwent previous surgical intervention (92.3% in proton cohort and 96.7% in photon cohort) as well as chemotherapy (65.4% in proton cohort, 80.0% in photon cohort, 10.6% surgery cohort; *p* = 0.0001). Detailed patient characteristics are displayed in Table 1 and Figure 1. Patients were assigned to three different therapy groups based on the main treatment modality, irrespective of previous therapy. Most patients in the radiation therapy groups had received additional surgery before undergoing radiation therapy.

### 2.2. Neuropsychological Assessment

Neuropsychological assessment included the following age standardized tests: Raven’s Coloured Progressive Matrices^©^ (CPM) and Raven’s Standard Progressive Matrices^©^ (SPM), Beery–Buktenica Developmental Test of Visuomotor Integration^©^ (VMI), Rey–Osterrieth Complex Figure Test^©^ (ROCF), Regensburger Word Fluency Test^©^ (WF), Wechsler Intelligence Scale for Children IV and Wechsler Adult Intelligence Scale WIE/WAIS-III^©^ (subtests working memory and processing speed (PS)) [15,16,17,18], therefore including performance in the domains of non-verbal intelligence (SPM), visuomotor integration (VMI), visuospatial construction (ROCF-C), and visuospatial memory (ROCF-R), categorial and lexical word fluency (WF-C, WF-L), working memory (WM), and processing speed (PS). Each of the outlined assessments was administered by a clinical neuropsychologist, enabling comprehensive identification of patients’ neuropsychological outcome and school performance. While testing was encouraged at four different time points prior to and following treatment, results were incomplete for some patients given inherent limitations in follow-up of patients referred to our center from far-reaching, disparate locations. 

### 2.3. Statistical Analysis

Normal distribution was ascertained with Shapiro–Wilk test and homogeneous variances via Levene’s test. A Student’s *t* test was performed to a level of significance of *p* < 0.05 for analyzing changes from baseline to follow-up. Intermodal group comparison between RT and surgery was analyzed using ANOVA. TOST analyses (two one-sided *t* tests) were performed to examine equivalence of treatment modalities with an equivalence margin set to ±15 IQ points. All test results were translated to an IQ scale. Differences in patient characteristics were analyzed with chi-square tests. Bonferroni correction was not utilized given the small patient population. Statistical analysis was performed using SPSS Statistics 25 (IBM^®^, New York, NY, USA).

### 2.4. Ethics Approval

This study was approved by the ethics committee of the University of Heidelberg, Germany (S-421/2015). The requirement of informed consent was waived by the ethical committee due to the retrospective nature of this study. All examinations and evaluations were performed following institutional guidelines and the Declaration of Helsinki of 1975 in its most recent and updated version.

## 3. Results

Results of longitudinal development after proton RT, photon RT, and surgery and intermodal group comparison are presented separately. Longitudinal analyses were conducted from date of initial consultation (baseline) to date of last follow-up (proton cohort: 42 months, photon cohort: 55 months, surgery: 55 months).

### 3.1. Proton RT: Longitudinal Development from Baseline to 42 Months after Proton RT

**Figure 2:**
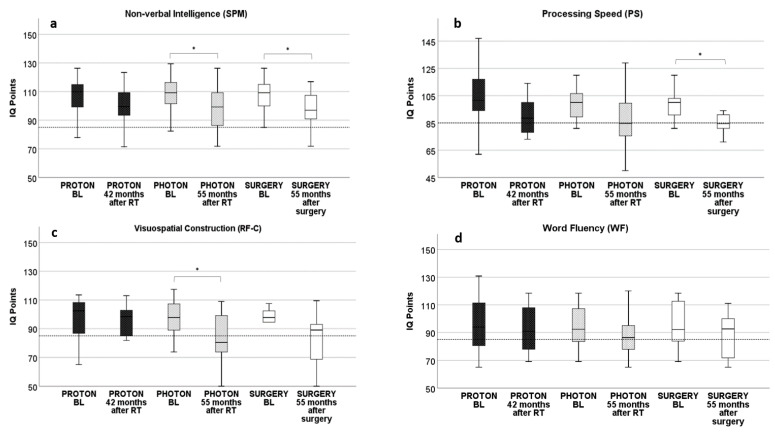
Neurocognitive test results of four main domains showing no significant difference in intermodal group comparison. Significant changes are indicated by *. Dotted line at 85 IQ points indicates a threshold below average values. Abbreviations: IQ = intelligence quotient, RT = radiation therapy, SPM = Standard Progressive Matrices, PS = processing speed, RF-C = Rey Figure for visuospatial construction, WF = word fluency. (**a**) Results of non-verbal intelligence showing significant decrease in longitudinal follow-up in the photon and surgery cohort; no differences in intermodal group comparison, all values above average (**b**) Results in processing speed showing significant decrease in longitudinal development in the surgery cohort with below average results in the surgery and photon cohort after 55 months of follow-up (**c**) Results of visuospatial construction with significant decrease in longitudinal follow-up in the photon cohort, mean results above average at any follow-up; no differences in intermodal group comparison (**d**) Results of word fluency with no significant alteration in longitudinal follow-up and no significant differences in intermodal group comparison.

Longitudinal neurocognitive assessment of the proton cohort from baseline to follow-up 42 months after treatment showed no alteration in any of the examined neurocognitive domains. Reporting the non-significant results, processing speed started with a baseline IQ of 103 and declined to an IQ of 91 after 42 months by 11.9% (*p* = 0.06) (Figure 2b). Visuomotor integration IQ started at 98 and showed a decline of 10.0% at 42 months, with an IQ of 89 (*p* = 0.05). Baseline IQ in working memory declined 3.9% from 97 to 94 (*p* = 0.48). Visuospatial memory was only mildly affected with a reduction of 4.3% (*p* = 0.59) from 96 to 91 after 42 months (Figure 2a). In the domain of visuospatial construction, baseline IQ was identical to follow-up IQ at 96 points (Figure 2c). Additionally, categorial and lexical word fluency showed minimal change at follow-up with categorial word fluency decreasing by 4.9% (from 95 to 91; *p* = 0.60) and lexical word fluency decreasing by 1.2% (from 92 to 91; *p* = 0.89) at 42 months after proton RT compared to baseline (Figure 2d). All these reported results in longitudinal development in the proton cohort showed no statistical significance (Table 2).

### 3.2. Photon RT: Longitudinal Development from Baseline to 55 Months after Photon RT

In the domain of non-verbal intelligence, there was a significant decline of 9.6% (98; *p* = 0.01) 55 months after photon irradiation compared to baseline (109) (Figure 2a). Visuospatial construction also showed a significant decline of 14.9% (*p* = 0.02), starting at 97 and declining to 82 after photon irradiation (Figure 2c). The other domains of processing speed, visuomotor integration, word fluency, working memory, and visuospatial memory showed no difference compared to baseline, even though a larger decline was seen in processing speed (10.4%; *p* = 0.12, Figure 2b) and visuomotor integration (9.4%; *p* = 0.06); however, performance was still above average after photon RT. Categorial and lexical word fluency were less affected, with the former declining by 4.4% (from 94 to 90; *p* = 0.67) and the latter by 1.6% (from 94 to 93; *p* = 0.76, Figure 2d). Visuospatial memory was seemingly unaffected by photon irradiation with baseline and follow-up IQ identical at 90. Except for a decline in non-verbal intelligence and visuospatial construction no statistical significance could be found in longitudinal development in the photon cohort (Table 2).

### 3.3. Surgery: Longitudinal Development from Baseline to 55 Months after Surgery

Long-term follow-up 55 months after surgery showed a significant decline in non-verbal intelligence by 10.7% (IQ 97; *p* = 0.01, Figure 2a). The largest decline was seen in processing speed, from a start of 99 to 89 after surgery, a decline of 14.9% (*p* = 0.002, Figure 2b). Also, there was a trend in visuospatial construction (15.1%; *p* = 0.09) with a decline from 96 to 81, with follow-up results below average (Figure 2c). Visuospatial memory showed no change at 89 at both baseline and follow-up. In terms of categorial and lexical word fluency, only a moderate decline was observed, 6.8% in the former (from 95 to 89; *p* = 0.31) and 5.0% in the latter (from 92 to 87; *p* = 0.42, Figure 2d). There was a decline of 6.9% in visuomotor integration from 94 to 90 points (*p* = 0.12). In summary, a significant decline could only be seen in the domains of non-verbal intelligence and processing speed, whereas all other described alterations showed no significance (Table 2).

### 3.4. Intermodal Comparison of Neurocognitive Results after RT and Surgery

Comparing neurocognitive performance of the three different treatment groups at approximately four years after intervention, there were differences, suggesting comparable performance among all groups. Fifty-five months after intervention, the RT (proton and photon) group showed slightly better outcomes in the domains of word fluency (−3.4% RT vs. −6.8% surgery; *p* = 0.54), visuospatial construction (−12.1% vs. −15.0%; *p* = 0.70), processing speed (−11.8% vs. −15.0%; *p* = 0.55) and non-verbal intelligence (−9.1% vs. −10.8%; *p* = 0.73). Visuomotor function was less affected by surgery (−6.8%) than by RT (−9.8%; *p* = 0.66). Equivalence analyses by TOST (two one-sided *t* tests) also identified statistically significant similarity, confirming that most neurocognitive outcomes were similar among different treatment groups. Non-verbal intelligence was equivalent at last follow-up with an IQ of 100, 42 months after proton RT; an IQ of 98, 55 months after photon RT; and an IQ of 97, 55 months after surgery (*p* = 0.01—significance equates to equivalence). Visuomotor integration showed equivalent results among photon RT (86) and surgery (88) 55 months after intervention (*p* = 0.005) and also after proton RT (89) compared to surgery (88) (*p* = 0.02). Processing speed (proton: 91; photon: 89; surgery: 84; *p* = 0.03) and working memory (proton: 94; photon: 94; surgery: 94; *p* = 0.002) were equivalent when comparing all three treatment groups at last follow-up. Word fluency, visuospatial construction, and visual memory were equivalent when comparing photon RT and surgery, whereas the proton cohort had better results. Categorial word fluency was equivalent between the photon and surgery cohorts, but not with the proton cohort (photon: 90, surgery: 89, *p* = 0.01; proton: IQ 91, *p* = 0.14). Similar results were seen in the domains of visuospatial construction (photon: IQ 82, surgery: IQ 81, *p* = 0.04; proton: IQ 96, *p* = 0.54) and visual memory (photon: IQ 90, surgery: IQ 89, *p* = 0.01; proton: IQ 91, *p* = 0.11), all showed equivalent results in a comparison of photon RT and surgery but not with the proton cohort. Lexical word fluency showed equivalent outcomes among proton and photon RT but was worse in the surgical cohort (proton: 91, photon: 93, *p* = 0.04; surgery: 87, *p* = 0.10) (Table 3). While there were no differences detected between the neurocognitive outcomes of the three treatment groups, equivalence analyses could show equivalent outcome results.

### 3.5. Scholastic Implications of Brain Tumor Treatment

**Figure 3:**
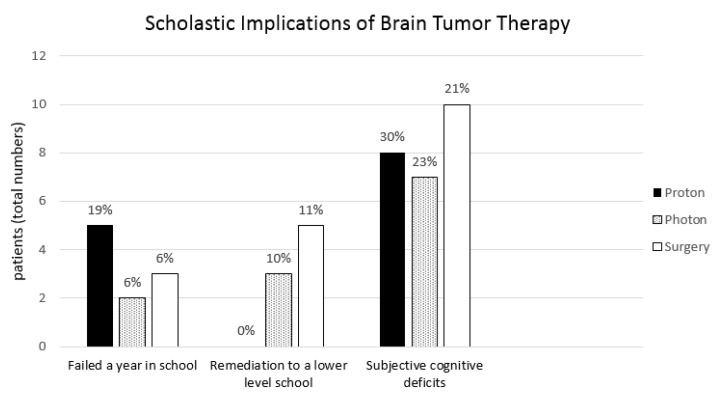
Scholastic implications of brain tumor therapy showing the results of all three therapy groups: proton, photon, and surgery cohort.

Regarding school performance, 19.7% in the proton group, 6.7% in the photon group, and 6.5% in the surgery group failed a year of school (*p* = 0.787). In the proton cohort no patient required remediation to a lower level due to poor performance, whereas 10.0% in the photon cohort and 11% in the surgery cohort had to change schools (*p* = 0.282). Subjective cognitive deficits that were noticed for the first time after completing brain tumor therapy, i.e., a lack of concentration or new problems with specific tasks like learning vocabulary, were reported by 26.8% in the RT cohort vs. 27.7% in the surgery group (*p* = 0.819). All children in this study received support from clinical neuropsychologists throughout therapy and follow-up. In total, 53% of patients received special remediation programs in school in close cooperation with teachers. Scholastic performance showed no difference when comparing radiation cohorts and surgery cohort (Figure 3).

### 3.6. Subgroup Analysis: Tumor Localization

One major aspect in neurocognitive outcomes can result from tumor localization. Therefore, we conducted a subgroup analysis of supra- and infratentorial tumor localization. Infratentorial tumor localization in our study showed impairment in the fields of processing speed, working memory, visuospatial construction, and verbal memory. The proton cohort showed significantly reduced processing speed in longitudinal follow-up in infratentorial tumor site compared to supratentorial tumor localization, with a reduction from an IQ of 111 to an IQ of 91 (*p* = 0.009). Also, working memory declined significantly in longitudinal follow-up after proton radiotherapy when treating infratentorial tumors, with a decline from an IQ of 99 to 83 (*p* = 0.007). In working memory of the proton cohort, direct comparison of the infratentorial with the supratentorial group showed significantly worse performance (*p* = 0.01). Working memory also showed a significant decline in longitudinal analysis of the photon cohort when tumors were located infratentorial in comparison to supratentorial, declining from IQ 110 to 90 (*p* = 0.01). Visuospatial construction showed significant decline in follow-up after proton RT with infratentorial tumors from an IQ of 109 to 89 (*p* = 0.01). Verbal memory in the surgery cohort showed significantly worse performance when tumors were located infratentorial in comparison to supratentorial (*p* = 0.007). The only neurocognitive domain that seemed to be more impaired by a supratentorial tumor site was visuomotor integration with a significant decline of performance in longitudinal follow-up after proton RT from IQ 103 to 85 (*p* = 0.02). The domains of non-verbal intelligence, word fluency, and visuospatial memory showed no differences between supratentorial and infratentorial tumor sites.

A further stratification in left- vs. right-sided tumors or tumors in specific brain areas was not feasible due to small patient numbers.

### 3.7. Subgroup Analysis: Extent of Tumor Resection

The extent of tumor resection differed in the different therapy groups. In order to stratify for possible bias, a comparison of gross total resection (GTR) and partial tumor resection (PTR) was conducted. Included in this analysis were all patients of the surgery cohort. Processing speed showed a better performance after PTR than GTR, with a difference of 18.6% (*p* = 0.009). Further longitudinal development reduced the difference between the two groups; at 55 months after surgery there was a slight difference of 6.4% in favor of PTR (*p* = 0.24). Word fluency showed a better result after PRT than GTR with a difference of 13.6% (*p* = 0.04) 19 months after surgery, but seemed to show no significant differences in further longitudinal follow-up (3.1% after 55 months, *p* = 0.73). In contrast, visuospatial construction performance was better after GTR by 14.8% (*p* = 0.02) two months after surgery but showed no differences in long-term follow-up. The domains of non-verbal intelligence, visuomotor integration, working memory, and visuospatial memory showed no differences between GTR and PTR. All the other domains showed temporary differences, but 55 months after surgery there were no differences to be found in any of the neurocognitive domains (Table 4).

## 4. Discussion

Our study showed equivalent outcomes in a variety of different neurocognitive domains of pediatric brain tumor patients treated with multimodal therapy approaches including radiation therapy and surgery in direct intermodal comparison to those treated with surgery alone. This is in contrast to the pervasive perception of the devastating impact of radiation therapy on the developing brain as previously reported [3,5]. Published results of more recent studies [19,20,21] indicated improved outcomes with increased conformality from modern radiation techniques by decreasing dose delivery to healthy brain tissue. Our findings support this theory and confirm similar outcomes after focal proton RT and surgery alone, similar to results recently published by Kahalley et al. [14]. Mounting evidence suggests improved neurocognitive outcome following treatment with modern conformal RT techniques, approaching those reported after neurosurgical intervention and underscoring the potential implications in altering treatment decision-making.

Examining the longitudinal development of neurocognitive domains after treatment, we found no decline after proton RT, but the domains of processing speed and visuospatial construction were compromised after photon RT, whereas processing speed and nonverbal intelligence declined following surgical intervention. Results among the proton cohort remained above average in all tested domains, supporting previously published findings [22,23,24]. Given the study design with large numbers of patients in radiation therapy groups having received additional surgery, results appear counterintuitive when only in the surgery cohort was a decline in follow-up detected. It seems likely that patient numbers were too small to reliably detect all significant differences. The proton RT group showed no decline in neurocognitive function in any of the tested domains but surgery alone seemed to compromise processing speed and nonverbal intelligence. It is probable that performance also declined in the proton cohort with additional surgery but might have been veiled by small patient numbers; conduction of follow-up assessment occurred approximately one year prior to the surgery cohort and included a group of solely irradiated patients in the proton cohort as small as 8%. Another possible explanation for the discrepancy between results in radiation cohorts receiving additional surgery and the described neurocognitive decline after surgery alone is that the treatment modality did not represent the largest impact on neurocognitive functioning. There might be other factors that influenced neurocognitive outcome after brain tumor therapy more than the surgery or radiation therapy itself, i.e., tumor localization, tumor type, or extent of tumor resection. Regarding tumor type, the inherent limitations in predetermined treatment categories made it difficult to pinpoint the influence on neurocognition of either tumor or treatment, i.e., high grade tumors might receive maximum multimodal therapy whereas low-grade tumors often undergo stepwise therapy escalation when needed.

Analyzing the neurocognitive outcome in regard to the extent of tumor resection, no differences between gross tumor resection and subtotal tumor resection could be seen in long-term follow-up 55 months after surgery. A temporarily better outcome could be found in processing speed and word fluency after subtotal tumor resection compared to gross total resection, whereas temporarily better outcomes were found in visuospatial construction after gross total resection. Other than implying a possible bias in comparison of different heterogeneous treatment groups, these results remain unclear. Temporarily better outcomes after partial tumor removal can be explained by less damage to healthy tissue due to the cautious resection. Temporarily better outcomes found in the domain of visuospatial construction after extensive tumor resection might be explained by reduction of tumor burden and mass effect. Since none those differences seemed to last in long-term follow-up, the clinical implications of these findings remain unclear and should be analyzed further in larger patient cohorts with homogeneous patient characteristics. In this analysis the surgery cohort included a slightly larger percentage of patients that underwent gross total resection than partial tumor resection, which might also account for the discrepancy in the detected decline of neurocognitive function after surgery alone vs. the stable results in radiation groups.

Regarding tumor localization, a separate analysis with larger patient cohorts might elucidate the pending questions. In our retrospectively assessed data with small patient cohorts we found worse neurocognitive outcomes in the domains of processing speed, working memory, visuospatial construction, and verbal memory in patients with infratentorial tumor sites compared to supratentorial tumor localization, especially in the radiation therapy groups. Only visuomotor integration showed a worse outcome in supratentorial tumor localization. Tumor localization was equally distributed in the three therapy groups, with approximately half of the patients with infratentorial and the other half with supratentorial tumor localization. This might also explain why no differences were found in the proton and photon cohorts in longitudinal analysis, when there was a decline in functioning in the surgery cohort. Since tumor localization is known as an important factor in neurocognitive functioning it is a relevant aspect that needs special attention. Nonetheless, due to the equal distribution of tumor localization in the therapy groups, a possible bias in comparison of the three groups might have accounted for this. The separate analysis of outcomes stratified for tumor localization underscores the fact that some domains like processing speed or visuospatial construction, which are largely dependent on intact white matter, are vulnerable and seem to be impacted by brain tumor therapy.

Similar to previous reports [25,26] the most vulnerable domain in the present analysis was processing speed, showing a decrease in longitudinal development with a below-average IQ in both the photon and surgery cohorts. Results were equivalent in the intermodality comparison, showing that processing speed is vulnerable independent of treatment modality. Processing speed is one of the major underlying domains that forms the foundation for many other neurocognitive functions dependent on intact white matter connections. While white matter tracts are particularly susceptible to radiation-induced impairment, brain surgery was also found to alter white matter functionality [27,28,29]. Due to the similar pathophysiological effect of white matter structures independent of the actual cause, we hypothesize that localized damage to white matter tracts rapidly impacts overall processing speed to the same extent, irrespective of treatment approach: radiation therapy or surgery. Similar results were seen in the domains of visuoconstruction and visuomotor integration, domains largely dependent on white matter connections, i.e., visuomotor integration dependent on integration of motor cortices and visual perception [30] or visuoconstruction dependent on connections between prefrontal cortices and parieto-occipital lobes [31,32,33]. In contrast, visual memory and word fluency, largely dependent on hippocampal structures [31,34,35,36,37], were not affected by modern brain tumor treatment. Whether this is caused by hippocampal sparing, common practice when possible in radiation treatment, or is explained by less dependence on intact white matter remains unclear.

Objective neuropsychological testing and scholastic performance showed comparable results among all tested domains. Nonetheless, approximately one-quarter to one-third of patients reported subjective neurocognitive deficits after completion of therapy, highlighting that decline of certain neurocognitive domains like processing speed might affect overall performance more than test results suggest. This relationship underscores the importance of key neurocognitive domains and, by extension, the integrity of white matter structures, which are known to be especially vulnerable in the pediatric population since myelination is not complete until late childhood. Despite a larger percentage of children reporting worse neurocognitive function after therapy, most children were able to continue class and achieve their aspired degree. This likely reflects the neuroplasticity of the pediatric brain but might also be attributed to the clinical neuropsychologists assigned to patients throughout therapy, with special training as well as rehabilitation programs provided to patients in collaboration with their teachers. As shown by multiple studies [38,39], damage of white matter tracts can to some extent be compensated by continuous training. Even structural alteration can be observed, i.e., increase in fiber myelination or reorganization of white matter tracts and fiber networks. Given the assumption that white matter destruction is the underlying mechanism for neurocognitive decline after therapy, these studies show the molecular and morphologic effects of specialized neurocognitive training, emphasizing the importance of close neuropsychological co-treatment. Compared to the general population, approximately 9.7% of the whole patient cohort had to repeat a class, suggesting better results than previously reported by early studies, i.e., Hoppe-Hirsch et al. describing scholastic problems in more than 80% of pediatric brain tumor patients after treatment [5] especially when compared to the lifetime prevalence of class repetition of the general population in Germany, which is estimated at 17%.

Limitations of this study include the small patient numbers and the retrospective design with limited follow-up in some patients and heterogeneous patient characteristics that might cause a bias in results as previously discussed, i.e., for tumor localization or extent of surgery. An additional limitation in the present analysis is inherent in the role of radiation treatment as an adjunct for patients who have disease progression: over 90% of patients in the RT groups underwent additional surgery and thus had a significantly larger treatment burden compared to patients who underwent surgery alone. Despite this significant increase in treatment burden, it is even more remarkable that outcomes are relatively equivalent among treatment groups. Due to these additional therapies in the radiation groups, a direct comparison between radiation therapy alone (proton or photon) and surgery alone was not feasible. Results rather suggest no further neurocognitive decline after adding radiation therapy to therapy regimes. Despite these limitations this study adds to the previously published thesis that modern radiation therapy has less impact than at first expected and that there are multifaceted influences on neurocognitive outcomes in pediatric brain tumor treatment that have to be taken into account. After detailed neuropsychological testing we could see more impact on domains dependent on white matter rather than on other brain structures, especially the hippocampal area. To this point, only a few such detailed tests after different modalities of pediatric brain tumor therapy have been reported. If continued efforts in prospective studies can further exculpate radiation therapy as a primary risk factor for neurocognitive decline, this might alter treatment decision-making, i.e., in low-grade tumors.

## 5. Conclusions

Considering the results of neurocognitive testing, our study shows no significant neurocognitive decline in the long-term follow-up of pediatric brain tumor patients after proton radiotherapy. We did not observe any significant differences in neurocognitive outcome after adding radiation therapy to surgery, compared to surgery alone, instead observing equivalent results among all treatment groups. This suggests that modern radiation therapy does not seem to affect cognition as much as described in the past. Domains dependent on intact white matter structures appear to be especially vulnerable to brain tumor treatment independent of treatment modality, but most patients were able to function in daily and scholarly life with only mild impairment.

## Figures and Tables

**Table 1:** Patient characteristics. Patient cohort with neurocognitive assessment in proton, photon, and surgery cohort.

Characteristics	Specification	Proton RT *n* = 26	Photon RT *n* = 30	Surgery *n* = 47
Gender	male (%)	16 (61.5)	15 (50.0)	25 (53.2)
	female (%)	10 (38.5)	15 (50.0)	22 (46.8)
Follow-up (in years)	after initial diagnosis (range)	6.6 (2.0–17.2)	9.2 (3.4–16.8)	6.3 (0.7–14.2)
Alive at last follow-up	yes (%)	23 (88.5)	28 (93.3)	44 (93.6)
	no (%)	3 (11.5)	2 (6.7)	3 (6.4)
Age at Intervention (in years)	mean (range)	9.4 (3.2–19.0)	9.6 (2.3–17.3)	10.5 (1.6–17.9)
Diagnosis	glioma (%)	11 (42.3) *^,†^	8 (26.7) ^†^	42 (89.4) *^,†^
	medulloblastoma (%)	2 (7.7)	13 (43.3)	0 (0)
	ependymoma (%)	5 (19.2)	3 (10.0)	1 (2.1)
	craniopharyngeoma (%)	1 (3.8)	0 (0)	3 (6.4)
	germinoma (%)	3 (11.5)	4 (13.3)	0 (0)
	others (%)	4 (15.4)	2 (6.7)	1 (2.1)
Grading	low-grade (WHO °I & °II)	12 (46.1) *	6 (20.0) ^†^	39 (83.0) *^,†^
	high grade (WHO °III & °IV)	10 (38.5) *	18 (60.0) ^†^	4 (8.5) *^,†^
	unknown	4 (15.4)	6 (20)	4 (8.5)
Localization	supratentorial (%)	15 (57.7)	14 (46.7)	23 (48.9)
	infratentorial (%)	9 (34.6)	16 (53.3)	21 (44.7)
	extraaxial (%)	2 (7.7)	0 (0)	3 (6.4)
Radiation Therapy	irradiation (%)	26 (100), proton	30 (100), photon	0 (0)
	total dose, mean (range) in Gy (RBE)	51.3 (16.0–74.0)	53.3 (30–68)	0
	PTV, mean in ccm (range)	262.9 (27.7–1691.4)	463.13 (13.05–4078.9)	0
	CTV, mean in ccm (range)	205.9 (16.1–1465.6)	46.5 (10.99–166.93)	0
	Total craniospinal irradiation	1 (3.8) **	14 (46.7) **	0 (0)
Chemotherapy	yes (%)	17 (65.4) *	24 (80.0) ^†^	5 (10.6) *^,†^
	no (%)	9 (34.6)	6 (20.0)	42 (89.4)
Surgery	no (%)	2 (7.7)	1 (3.3)	0 (0)
	STR (%)	15 (57.7)	15 (50.0)	21 (44.7)
	GTR (%)	9 (34.6)	14 (46.7)	26 (55.3)

*n* = number of, e.g., patients; RT = proton irradiation; FU = follow up; ° = grade; WHO = brain tumor classification established by the World Health Organization; Gy(RBE) = Gray, relative biological effectiveness accounting for proton irradiation; PTV = planning target volume; CTV = clinical target volume; ccm = cubic centimeter; STR = subtotal resection; GTR = gross total resection. * statistically significant difference between proton radiotherapy cohort and surgery cohort; ^†^ statistically significant difference between photon radiotherapy cohort and surgery cohort; ** statistically significant difference between proton radiotherapy cohort and photon radiotherapy cohort.

**Table 2:** Neurocognitive testing from baseline to latest follow-up, organized by domain and treatment modality.

Domains	Specification	Proton (Follow-Up 42 Months after RT)	Photon (Follow-Up 55 Months after RT)	Surgery (Follow-Up 55 Months after Surgery)
non-verbal intelligence (CPM/SPM)	baseline (IQ-points)	107.2	108.5	108.3
	follow-up (IQ-points)	99.9	98.1	96.6
	change to baseline	−6.8% (*p* = 0.15)	−9.6% (***p* = 0.01**)	−10.7% (***p* = 0.01**)
visuomotor integration (VMI)	baseline (IQ-points)	98.4	95.2	94.3
	follow-up (IQ-points)	88.6	86.3	89.9
	change to baseline	−9.9% (*p* = 0.05)	−9.4% (*p* = 0.06)	−6.9% (*p* = 0.12)
word fluency (WF-C) categorial	baseline (IQ-points)	95.1	94.0	95.2
	follow-up (IQ-points)	91.2	89.8	88.7
	change to baseline	−4.1% (*p* = 0.60)	−4.4% (*p* = 0.67)	−6.8% (*p* = 0.31)
word fluency (WF-L) lexical	baseline (IQ-points)	92.0	94.2	91.5
	follow-up (IQ-points)	91.0	92.6	86.9
	change to baseline	−1.0% (*p* = 0.89)	−1.6% (*p* = 0.76)	−5.0% (*p* = 0.42)
processing speed (PS)	baseline (IQ-points)	102.7	99.2	99.2
	follow-up (IQ-points)	90.5	88.9	84.4
	change to baseline	−11.9% (*p* = 0.06)	−10.4% (*p* = 0.12)	−14.9% (***p* = 0.002**)
working memory (WM) (digit span)	baseline (IQ-points)	97.7	99.1	97.6
	follow-up (IQ-points)	93.9	93.6	93.5
	change to baseline	−3.8% (*p* = 0.48)	−5.6% (*p* = 0.19)	−4.3% (*p* = 0.53)
visuospatial construction (ROCF-C)	baseline (IQ-points)	95.93	96.5	95.6
	follow-up (IQ-points)	95.9	82.1	81.2
	change to baseline	−0.03% (*p* = 0.99)	−14.9% (***p* = 0.02**)	−15.1% (*p* = 0.09)
visuospatial memory (ROCF-R)	baseline (IQ-points)	95.5	89.84	88.9
	follow-up (IQ-points)	91.4	89.78	89.1
	change to baseline	−4.3% (*p* = 0.59)	−0.1% (*p* = 0.99)	+0.20 (*p* = 0.98)

RT = radiotherapy, CPM = Raven’s Coloured Progressive Matrices, SPM = Raven’s Standard Progressive Matrices, VMI = visuomotor integration, WF-C = categorial word fluency, WF-L = lexical word fluency, PS = processing speed, WM = working memory, ROCF-C = Rey Figure Copy, ROCF-R = Rey Figure Recall. Boldface figures represent significant changes (*p* < 0.05).

**Table 3:** Results of equivalence analysis (TOST = two one-sided *t*-tests), statistical significance accounting for equivalent results.

Equivalence Analysis (Significance Equates to Equivalence)	Specification	Proton (Follow-Up 42 Months after RT)	Photon (Follow-Up 55 Months after RT)	Surgery (Follow-Up 55 Months after Surgery)
non-verbal intelligence (CPM/SPM)	follow up (IQ-points)	99.9	98.1	96.6
	difference (equivalence) to proton	-	−1.8 (***p* = 0.01**)	−3.3 (***p* = 0.01**)
	difference (equivalence) to photon	+1.8 (*p* = 0.01)	-	−1.5 (***p* = 0.002**)
	difference (equivalence) to surgery	+3.3 (*p* = 0.01)	+1.5 (***p* = 0.01**)	-
visuomotor integration (VMI)	follow-up (IQ-points)	88.6	86.3	89.9
	difference (equivalence) to proton	-	−2.3 (*p* = 0.09)	−0.7 (***p* = 0.02**)
	difference (equivalence) to photon	+2.3 (*p* = 0.09)	-	+1.6 (***p* = 0.005**)
	difference (equivalence) to surgery	+0.7 (*p* = 0.02)	−1.6 (***p* = 0.005**)	-
word fluency (WF-C) categorial	follow-up (IQ-points)	91.2	89.8	88.7
	difference (equivalence) to proton	-	−1.4 (*p* = 0.05)	−2.5 (*p* = 0.149)
	difference (equivalence) to photon	+1.4 (*p* = 0.05)	-	−1.2 (***p* = 0.01**)
	difference (equivalence) to surgery	+2.5 (*p* = 0.149)	+1.2 (***p* = 0.01**)	-
word fluency (WF-L) lexical	follow-up (IQ-points)	91.0	92.6	86.9
	difference (equivalence) to proton	-	+1.6 (***p* = 0.04**)	−4.1 (*p* = 0.07)
	difference (equivalence) to photon	−1.6 (*p* = 0.04)	-	−5.7 (*p* = 0.10)
	difference (equivalence) to surgery	+4.1 (*p* = 0.07)	+5.7 (*p =* 0.10)	-
processing speed (PS)	follow-up (IQ-points)	90.5	88.9	84.4
	difference (equivalence) to proton	-	−1.6 (***p* = 0.04**)	−6.1 (***p* = 0.03**)
	difference (equivalence) to photon	+1.6 (*p* = 0.04)	-	−4.5 (*p* = 0.11)
	difference (equivalence) to surgery	+6.1 (*p* = 0.03)	+4.5 (*p* = 0.11)	-
working memory (WM) (digit span)	follow-up (IQ-points)	93.9	93.6	93.5
	difference (equivalence) to proton	-	−0.3 (***p* = 0.005**)	−0.4 (***p* = 0.03**)
	difference (equivalence) to photon	+0.3 (***p* = 0.005**)	-	−0.1 (***p* = 0.002**)
	difference (equivalence) to surgery	+0.4 (***p* = 0.03**)	+0.1 (***p* = 0.002**)	-
visuospatial construction (ROCF-C)	follow-up (IQ-points)	95.9	82.1	81.2
	difference (equivalence) to proton	-	−13.8 (*p* = 0.49)	−14.7 (*p* = 0.54)
	difference (equivalence) to photon	+13.8 (*p* = 0.49)	-	−0.9 (***p* = 0.04**)
	difference (equivalence) to surgery	+14.7 (*p* = 0.54)	+0.9 (***p* = 0.04**)	-
visuospatial memory (ROCF-R)	follow-up (IQ-points)	91.4	89.8	89.1
	difference (equivalence) to proton	-	−1.6 (*p* = 0.12)	−2.3 (*p* = 0.11)
	difference (equivalence) to photon	+1.6 (*p* = 0.12)	-	−0.7 (***p* = 0.01**)
	difference (equivalence) to surgery	+2.3 (*p* = 0.11)	+0.7 (***p* = 0.01**)	-

RT = radiotherapy, CPM = Raven’s Coloured Progressive Matrices, SPM = Raven’s Standard Progressive Matrices, VMI = visuomotor integration, WF-C = categorial word fluency, WF-L = lexical word fluency, PS = processing speed, WM = working memory, ROCF-C = Rey Figure Copy, ROCF-R = Rey Figure Recall. Boldface figures represent significant equivalence (*p* < 0.05).

**Table 4:** Results of subgroup analysis. Comparison of neurocognitive outcome in eight different neurocognitive domains after partial tumor resection (PTR) versus gross total resection (GTR) in longitudinal follow-up.

Surgery Cohort	Specification	2 Months after Surgery	2 Months after Surgery	19 Months after Surgery	19 Months after Surgery	32 Months after Surgery	32 Months after Surgery	55 Months after Surgery	55 Months after Surgery
		PTR	GTR	PTR	GTR	PTR	GTR	PTR	GTR
non-verbal	IQ points	105.7	105.6	103.6	102.5	104.5	100.5	104.1	92.1
intelligence	difference PTR & GTR	+0.1% (*p* = 0.99)	−0.1% (*p* = 0.99)	+1.1% (*p* = 0.84)	−1.1% (*p* = 0.84)	+3.8% (*p* = 0.42)	−3.8% (*p* = 0.42)	+11.5% (*p* = 0.07)	−11.5% (*p* = 0.07)
visuomotor	IQ points	88.7	97.3	90.1	95.5	90.3	92.2	91.0	86.0
integration (VMI)	difference PTR & GTR	−9.7% (*p* = 0.34)	+9.7% (*p* = 0.34)	−6.0% (*p* = 0.19)	+6.0% (*p* = 0.19)	−2.1% (*p* = 0.63)	+2.1% (*p* = 0.63)	+5.5% (*p* = 0.39)	−5.5% (*p* = 0.39)
word fluency	IQ points	88.9	92.8	93.5	89.1	96.5	87.4	90.1	87.8
categorial	difference PTR & GTR	-4.4% (*p = 0.72*)	+4.4% (*p* = 0.72)	+4.7% (*p* = 0.51)	−4.7% (*p* = 0.51)	+9.4% (*p* = 0.11)	−9.4% (*p* = 0.11)	+2.6% (*p* = 0.81)	−2.6% (*p* = 0.81)
word fluency	IQ points	96.0	91.3	99.4	85.9	96.1	88.5	88.4	85.6
lexical	difference PTR & GTR	+4.9% (*p* = 0.62)	−4.9% (*p* = 0.62)	+13.6% (***p* = 0.04**)	−13.6% (***p* = 0.04**)	+7.9% (*p* = 0.23)	−7.9% (*p* = 0.23)	+3.1% (*p* = 0.73)	−3.1% (*p* = 0.73)
processing speed	IQ points	109.3	89.0	100.4	90.3	96.8	92.9	87.8	82.2
	difference PTR & GTR	+18.6% (***p* = 0.009**)	−18.6% (***p* = 0.009**)	+10.1% (*p* = 0.09)	−10.1% (*p* = 0.09)	+4.0% (*p* = 0.49)	−4.0% (*p* = 0.49)	+6.4% (*p* = 0.24)	-6.4% (*p* = 0.24)
working memory	IQ points	115.5	101.4	100.8	98.9	103.0	99.3	91.3	94.4
(digit span)	difference PTR & GTR	+12.2% (*p* = 0.15)	−12.2% (*p* = 0.15)	+2.0% (*p* = 0.78)	−2.0% (*p* = 0.78)	+3.6% (*p* = 0.60)	−3.6% (*p* = 0.60)	−3.4% (*p* = 0.74)	+3.4% (*p* = 0.74)
visuospatial	IQ points	90.7	104.1	84.7	92.7	84.0	90.9	76.5	85.2
construction	difference PTR & GTR	−14.8% (***p* = 0.02**)	+14.8% (***p* = 0.02**)	−9.4% (*p* = 0.42)	+9.4% (*p* = 0.42)	−8.2% (*p* = 0.44)	+8.2% (*p* = 0.44)	−11.4% (*p* = 0.54)	+11.4% (*p* = 0.54)
visuospatial	IQ points	81.0	100.6	90.3	98.3	87.4	92.9	92.1	86.5
memory	difference PTR & GTR	−24.2% (*p* = 0.05)	+24.2% (*p* = 0.05)	−8.9% (*p* = 0.25)	+8.9% (*p* = 0.25)	−6.3% (*p* = 0.37)	+6.3% (*p* = 0.37)	+6.1% (*p* = 0.50)	−6.1% (*p* = 0.50)

PTR = partial tumor resection, GTR = gross total resection, IQ = intelligence quotient. Boldface figures represent significant equivalence (*p* < 0.05).

## Data Availability

The data presented in this study is available within the article.
